# Case report: Metastatic Merkel cell carcinoma presenting seven years after loco-regional disease resection of primary tumor with interval in-transit and nodal metastases

**DOI:** 10.3389/fonc.2023.1217816

**Published:** 2023-07-05

**Authors:** Joshua Rusheen, James Clune, Stephan Ariyan, Raymond Baumann, Harriet Kluger, Kelly Olino, Sarah Weiss

**Affiliations:** ^1^ Department of Medicine, Yale University School of Medicine, New Haven, CT, United States; ^2^ Section of Plastic and Reconstructive Surgery, Yale University School of Medicine, New Haven, CT, United States; ^3^ Database Management, Yale University School of Medicine, New Haven, CT, United States; ^4^ Section of Medical Oncology, Yale University School of Medicine, New Haven, CT, United States; ^5^ Department of Surgery, Section of Surgical Oncology, Yale University School of Medicine, New Haven, CT, United States; ^6^ Department of Medical Oncology, Rutgers Cancer Institute of New Jersey, New Brunswick, NJ, United States

**Keywords:** Merkel cell carcinoma, Merkel cell, In-transit, surveillance, recurrence

## Abstract

Merkel cell carcinoma (MCC) is a rare tumor with a high risk of recurrence after definitive therapy; however, the optimal duration of surveillance is unclear. First recurrences typically occur within 3 years. National guidelines recommend that patients undergo physical examination and imaging for surveillance during this time period. However, the duration of surveillance beyond this is not defined. Here, we describe a case of a patient developing a recurrence of MCC 7 years after the primary diagnosis with interval in-transit and regional lymph node metastases 15 months following the treatment of the primary MCC. Such late recurrences are rare, largely not reported, and the risk factors contributing to late recurrences are not well described. This case highlights the possibility of late recurrences of MCC after an initial in-transit and nodal recurrence and underscores the importance of identifying predictors of recurrence that may better guide the duration of surveillance.

## Introduction

Merkel cell carcinoma (MCC) is a rare and aggressive neuroendocrine tumor with an incidence of 1,972 cases per year in the United States that carries a high risk of recurrence and/or metastasis (1). MCC was originally called trabecular carcinoma of the skin because the cells were composed of solid trabeculae and lacked acini (2). However, the term Merkel cell carcinoma was later coined because with additional histologic characterization, the cells resembled Merkel cells from the basal layer of the epidermis with the assumption that it originated from them (3, 4). MCC characteristically presents as a red-violet cutaneous nodule on sun-exposed skin in elderly individuals with a median age of 75–80 years. Risk factors include fair skin, immunosuppression, and history of hematologic and/or cutaneous malignancies (4–8). The standard of care for primary MCC is wide local surgical excision and sentinel lymph node (SLN) biopsy. Adjuvant radiation is administered to primary tumors with high-risk features and to regional lymph nodes in patients with a positive SLN biopsy and/or after completion of lymph node dissection for which the pathology shows multiple involved nodes and/or extracapsular extension. Active surveillance with physical exam and imaging is the standard after definitive therapy for MCC; however, adjuvant immunotherapy with PD-1 inhibition is under active investigation in clinical trials (9–11).

The optimal duration of clinical and imaging surveillance for patients with a history of definitively treated MCC is unclear. The National Comprehensive Cancer Network (NCCN) guidelines v2.2022 recommend physical examination every 3–6 months for 3 years, followed by every 6–12 months without a specified end date, with imaging recommended as “clinically indicated,” particularly for high-risk patients. In a recent large cohort study from an MCC data repository, approximately 95% of first MCC recurrences occurred within 3 years of diagnosis (12). Late recurrences are rare. Statistics on late recurrences or second recurrences for MCC, as we report here, are largely absent from the literature and are rarely recorded in clinical databases. Here, we report a case of a patient with MCC of the left calf who developed a late recurrence 7 years after the primary diagnosis and after an initial in-transit and regional nodal recurrence. We highlight the importance of ongoing awareness for MCC recurrence beyond 3 years and discuss clinical and emerging laboratory factors that may better identify those at higher risk.

## Case

A woman in her 80s presented to her dermatologist after developing a 2-mm nodular lesion on the left calf that had been present for 2 months. She had a history of extensive sun exposure as well as history of a cutaneous squamous cell carcinoma of the forehead and basal cell carcinoma of the forehead and right medial canthus. She had no history of immunosuppression and no family history of MCC or other skin cancers. Biopsy of the lesion was performed, and pathology showed an intraepithelial proliferation of atypical epithelioid cells with pale chromatin, a high nuclear to cytoplasmic ratio, and mitotic activity. Immunohistochemical staining was positive for pan-cytokeratin (MNF116) and dot-like perinuclear staining with cytokeratin (CK) 20 and negative for S100. TTF-1 was not performed. She subsequently underwent wide excision for which pathology showed a 2-mm-thick, nodular growth pattern MCC, with greatest tumor dimension of 2 mm. Peripheral and deep margins were negative. There was no lymphovascular invasion and mitotic rate was 5 per high power field. Two left inguinal SLNs and one left pelvic SLN were negative by H&E examination and for CK20. The pelvic lymph node was also negative for AE1/AE3. Merkel cell polyomavirus (MCPyV) was not tested. Staging CT of the chest, abdomen, and pelvis was negative for distant disease. Final staging was pT1N0M0, stage IA, and the patient subsequently was followed on surveillance every 6 months.

Fifteen months later, she developed an in-transit recurrence at the medial left thigh confirmed via punch biopsy. She then underwent a wide local excision and SLN biopsy which showed isolated cells in two out of four left inguinal lymph nodes. Whole-body PET-CT was negative for distant metastasis, and her MCC was upstaged to pT1N3M0, stage IIIB. She underwent postoperative radiation therapy with 5,000 cGy to the left groin in 25 fractions, 5,000 cGy to the left thigh in 25 fractions, and 5,000 cGy to the left calf in 25 fractions. Subsequently, she continued on surveillance and underwent annual CT of the chest, abdomen, and pelvis.

**Figure 1:**
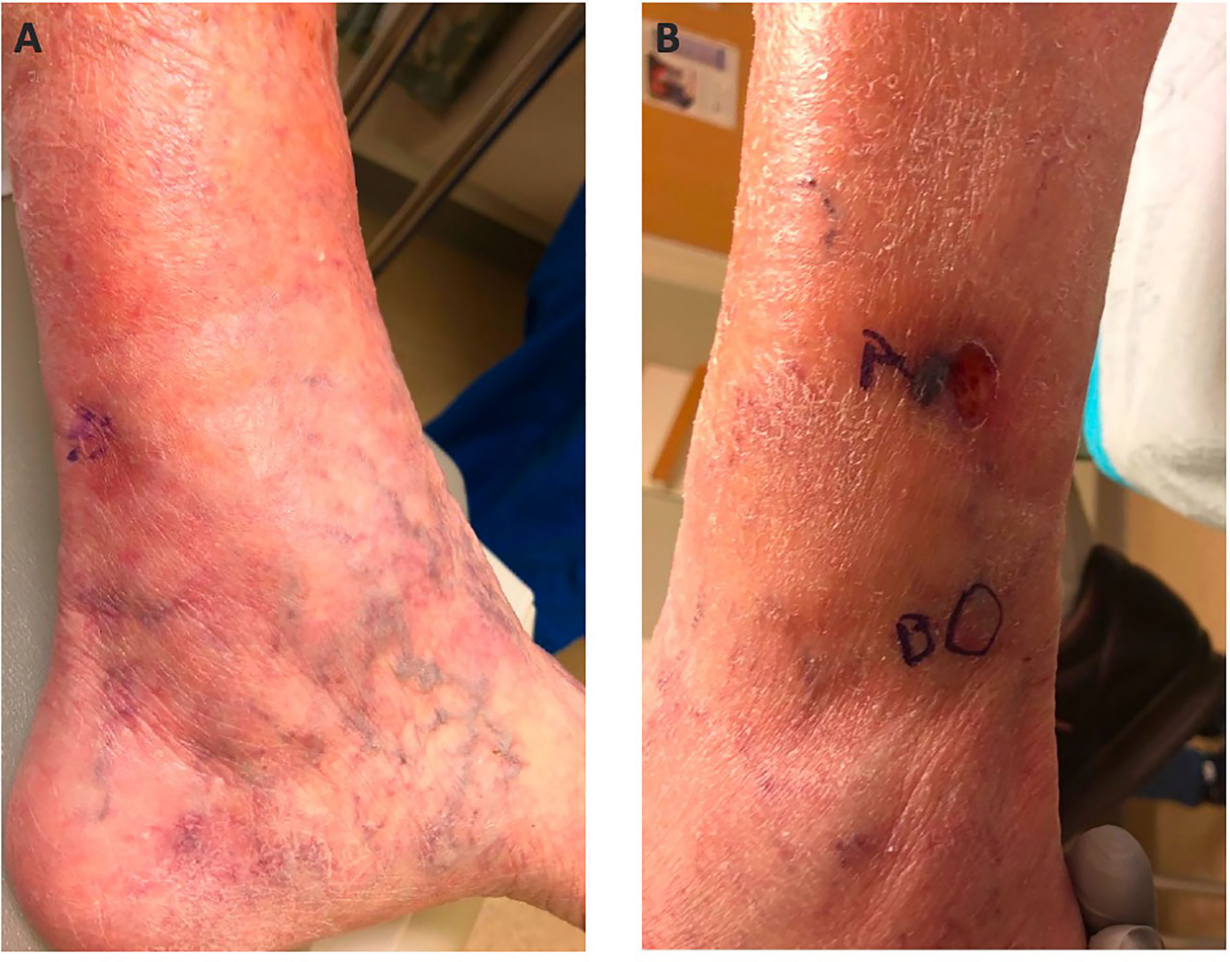
Medial Ankle **(A)** and Lateral Ankle **(B)**.

Just over 7 years (86 months) after the primary diagnosis, the MCC recurred distal to the primary site. Three separate metastatic lesions were biopsied and were positive for MCC (**Figure 1**). PET-CT was negative for other sites of disease or distant metastases. Due to the multifocal nature of the recurrence, surgical resection was not favored, and pembrolizumab 200 mg IV every 3 weeks was initiated. Treatment was continued for 1 year and resulted in a complete response (CR). At the time of this publication, she remains in CR and continues on close surveillance with body imaging every 4 months.

## Discussion

We describe an 80-year-old woman with a history of a stage IA MCC of the left calf who developed an in-transit and regional nodal recurrence 15 months after initial diagnosis, followed by multifocal metastatic recurrence distal to the primary site 7 years after initial diagnosis. This case is notable for a late MCC metastasis, which is rarely described, and raises the questions of how long patients with MCC should undergo surveillance and the factors that might contribute to the risk of recurrence.

A recent study examined the rate of first MCC recurrence from a large Seattle data repository collecting information on MCC patients from 2003 to 2019. Of 618 MCC patients, the 5-year recurrence rate was 40%, and 95% of recurrences arose within the first 3 years (12). Beyond 3 years, the recurrence-free survival curves plateau among all stages of MCC, including older studies that pre-dated use of anti-PD-1/PD-L1, as well as from data from our institution (12–22). The Yale Cancer Center MCC registry includes data from 216 MCC patients collected from 1996 to 2022. The majority of these patients had stage I–III MCC at diagnosis and age ranged from 42 to 98. A total of 56 (26%) patients experienced a first recurrence, with 95% of first recurrences presenting within the first 2 years of MCC diagnosis. The latest first recurrence occurred at 3.4 years.

Factors that can identify MCC patients at the highest risk of recurrence are under active study. Based on the referenced data, the vast majority of treated MCC patients who have not had a recurrence within 3 years of diagnosis are unlikely to recur. Surveillance imaging and physical exam are recommended by national guidelines, but the duration of surveillance follow-up for these patients is unclear, with current NCCN guidelines recommending regular physical exams for up to 3 years and less frequent thereafter, whereas imaging is to be performed as “clinically indicated”. This is similar to SITC practice guidelines, except for recommendations of surveillance imaging to be performed for high-risk MCC every 3–6 months for 2 years followed by every 6–12 months until 5 years (23). Beyond 3–5 years, patients who have already recurred and those who are immunocompromised remain at a higher risk for recurrence and may warrant longer follow-up. Moreover, additional non-staging factors such advanced age and other medical comorbidities should be considered in tailoring a patient’s surveillance period beyond the initial 3–5 years. Based on this case, a first in-transit and/or nodal MCC recurrence should be considered a high-risk factor in the development of second and/or late MCC recurrence. There is a lack of available statistics on late or second MCC recurrences in the literature or in clinical databases.

Other factors may also merit longer surveillance periods. Merkel cell polyomavirus (MCPyV), a double-stranded DNA virus, has been associated with MCC oncogenesis. MCPyV-positive MCC is associated with significantly longer recurrence-free survival as compared with MCPyV-negative MCC and may warrant longer surveillance periods. Moreover, patients with rising titers of MCpyV oncoprotein antibodies during the surveillance period may also be monitored at shorter intervals or with sooner imaging evaluation (24, 25). However, it is unknown whether measures of MCPyV are useful to predict late recurrences. In addition, circulating tumor DNA (ctDNA) is an emerging biomarker that has been used to document evidence of minimal residual disease and early recurrence and could be used to track responses to systemic therapies across multiple tumor types including MCC. However, ctDNA has yet to be fully validated and incorporated into routine clinical practice for MCC. Several studies suggest that ctDNA levels may be sensitive enough to detect early recurrence in MCC and could potentially be used as a tool to monitor these patients for the longer term (26, 27). For example, in this case, ctDNA levels may have been used to follow for recurrence after surgical intervention. ctDNA can also be used in MCPyV-negative MCC as it can be independent of the tumor’s viral status.

This case report challenges the currently perceived 3-year timeframe of anticipated risk of MCC recurrence and surveillance recommendations for 3–5 years after resection of loco-regional disease. Strengths of this report include a detailed presentation of this patient’s clinical course, which highlights that the late recurrence here developed years after definitive treatment of a first in-transit and nodal recurrence. Limitations of this report include a lack of generalizability for MCC patients since there is little reported on late recurrences in the literature. However, we emphasize the need to record data on second and late MCC recurrences moving forward.

Improved prognostic biomarkers are needed in patients with MCC. As MCC is a rare tumor type, initiatives to pool patient data across institutions and countries to have access to larger data sets and tumor banks for further study of MCC are warranted. While a surveillance period of at least 3–5 years for patients with treated MCC is standard (23), it is still unclear how long to follow MCC patients thereafter and with what surveillance mechanisms. It is paramount that clinicians consider both the stage and non-staging factors when determining the appropriate duration of surveillance for patients, especially in-transit and nodal recurrences, which is likely a high-risk factor for both subsequent and late recurrences. ctDNA may emerge as an important and minimally invasive tool that could lend itself to longer-term follow-up when compared with repetitive imaging studies, for example, which over time incur cumulative radiation exposure to the patient and have financial implications for the healthcare system, although further study is needed. Regardless, patients with a diagnosis of MCC should be counseled that the risk of late MCC recurrence is low, but not impossible.

## Data availability statement

The raw data supporting the conclusions of this article will be made available by the authors, without undue reservation.

## Ethics statement

Written informed consent was obtained from the participant/patient(s) for the publication of this case report.

## Author contributions

JR, JC, SA, RB, HK, KO, and SW were all involved in the drafting and review of this report. JR, JC, SA, HK, KO, and SW were involved in the medical care described in this report. RB provided data and information from the center’s registry. All authors contributed to the article and approved the submitted version.
